# Assessment of visual processing in Alzheimer’s Disease and Lewy Body Dementia using Fast Periodic Visual Stimulation

**DOI:** 10.1002/alz70861_108591

**Published:** 2025-12-23

**Authors:** Oliver P Hermann, Karin Petrini, Elizabeth Coulthard, George Stothart

**Affiliations:** ^1^ University of Bath (The), Bath, Avon UK; ^2^ University of Bath, Bath UK; ^3^ University of Bristol, Bristol UK

## Abstract

**Background:**

It is vital that functional biomarkers of dementia are developed that can identify early cognitive change and stratify cognitive deficits in different aetiologies. Visuoperceptual impairment occurs to varying degrees in Lewy body disease (LBD) and Alzheimer’s Disease (AD) and its quantification provides an opportunity to delineate the two diseases. Fast periodic visual stimulation (FPVS) is an electroencephalographic (EEG) marker of discrimination between two classes of frequency‐tagged stimuli, that can be adapted to capture different cognitive functions. FPVS tasks provide a rapid (<3mins), objective measure of brain function, implicitly and passively, i.e. the participant is not required to respond or even understand the task. Here, we assess whether FPVS visuoperceptual tasks can differentiate between LBD, AD, and cognitively heathy older adults (HOA).

**Method:**

Six patients with LBD, nine with AD, and 40 age‐matched HOAs, completed FPVS tasks that implicitly measured different forms of visuoperception: orientation discrimination, pseudo‐object perception, real object recognition, and positional discrimination. Visuoperception was also assessed behaviourally using the Freiburg Visual Acuity and Contrast Test, and the Visual Object and Space Perception Battery (VOSP). Global cognitive functioning was assessed using the Montreal Cognitive Assessment (MoCA). Data collection is ongoing with target samples sizes of 20 per patient group.

**Result:**

Across FPVS tasks, implicit visuoperception was reduced in patients compared to HOAs, with greater impairment observed in LBD than in AD. Per task, the between group performance was: orientation discrimination, HOA > AD > LBD (η2 = .145, *p* =0.045); pseudo‐object perception, HOA > AD > LBD (η2 = .232, *p* =0.020); real object recognition, HOA > AD = LBD (η2 = .096, *p* =0.080); positional discrimination, HOA > AD > LBD (η2 = .085, *p* =0.220).

**Conclusion:**

FPVS implicitly captures different forms of visuoperception in cognitively healthy older adults and is sensitive to impairment in AD and LDB. Furthermore, the technique can delineate DLB from AD using measures of orientation discrimination, pseudo‐object perception and positional discrimination, potentially guiding differential diagnosis.

